# Supplementary material for: Functionalized graphene-oxide grids enable high-resolution cryo-EM structures of the SNF2h-nucleosome complex without crosslinking
Source: Nat Commun. 2024 Mar 12;15:2225. doi: 10.1038/s41467-024-46178-y (PMC10933330; doi:10.1038/s41467-024-46178-y)
Supplement: Supplementary file 1 — Supplementary Information [file 41467_2024_46178_MOESM1_ESM.pdf]

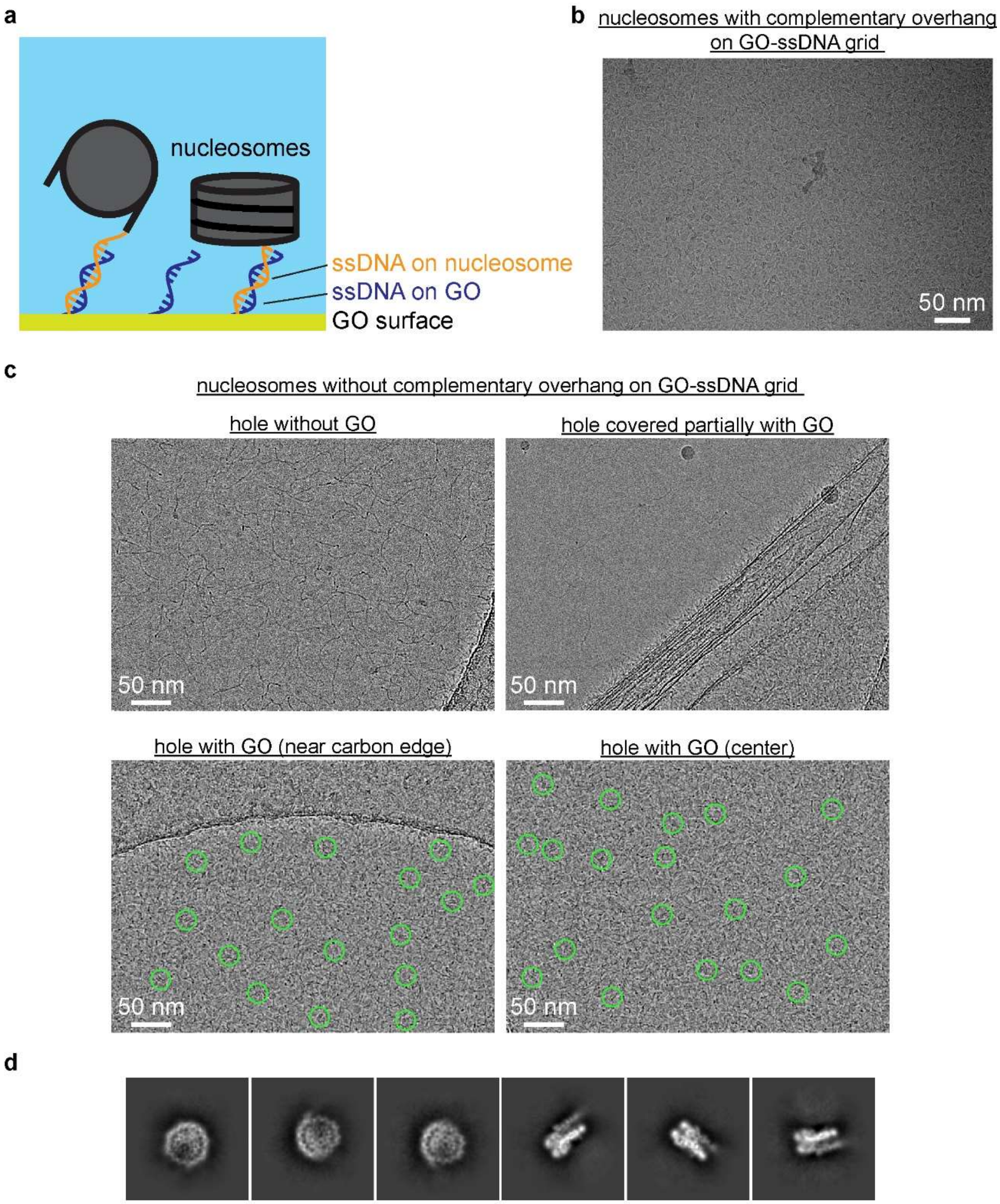

Supplementary Figure 1: ssDNA GO grids protect nucleosomes from the AWI.

a) Schematic illustrating the use of complementary DNA sequences to bring nucleosomes closer to the GO surface away from the AWI.
b) Representative micrograph of nucleosomes with complementary ssDNA overhang on a ssDNA GO grid. The experiment was performed once with several similar micrographs from a screening experiment.

c) Representative micrographs of nucleosomes on a ssDNA GO grid. The experiment was repeated multiple times (n > 3) with similar results. The number of micrographs collected varied for each experiment, ranging from ~10 for screening experiments to over 1,000 for a larger collection. Areas without GO display denatured nucleosomes (upper left). A hole partially covered with GO shows nucleosomes are only visible in the area with GO (upper right). The nucleosomes appear uniformly spread across holes covered with GO (lower left and lower right).

d) Representative 2D classes showing intact nucleosomes on a ssDNA GO grid.

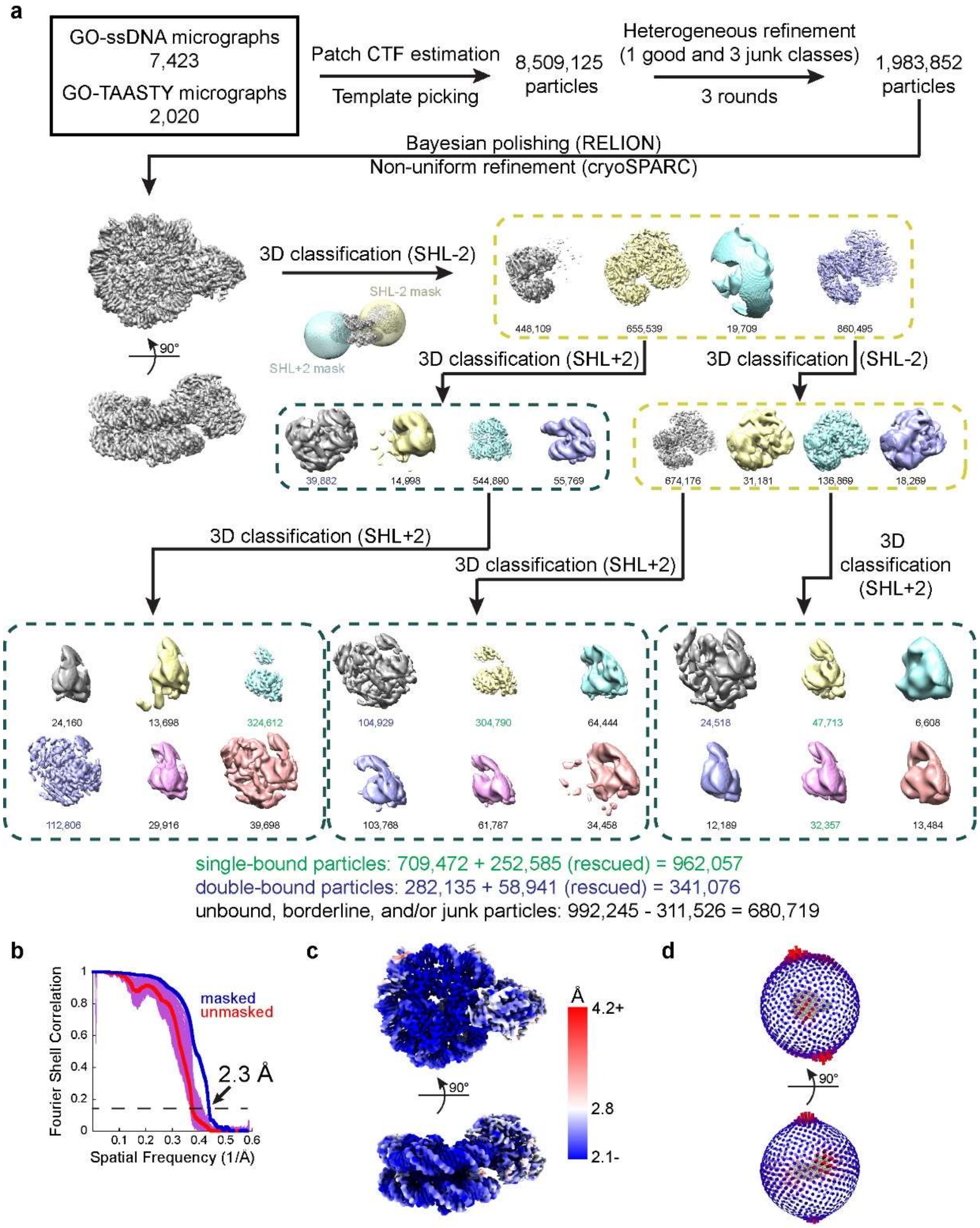

Supplementary Figure 2: Cryo-EM data processing for SNF2h-nucleosome datasets.

a) Schematic illustrating the initial processing workflow from micrographs to separating single- and double-bound SNF2h-nucleosome complexes as described in Methods. Particle counts for single-bound classes are colored in green and particle counts for double-bound classes are colored in blue. An additional round of classification (not graphically depicted) with particles from other classes was performed to rescue additional single- and double-

bound particles (252,585 and 58,941 particles, respectively), leading to the final particle counts denoted.

b) Gold-standard FSCs for the consensus map containing both single- and double-bound particles determined without masking (red) and by cryoSPARC masking (blue), as well as directional FSCs determined without masking (magenta).

c) The consensus SNF2h-nucleosome map surface colored by local resolution determined by cryoSPARC with FSC cutoff of 0.143.

d) Angular distribution plots for the consensus SNF2h-nucleosome map.

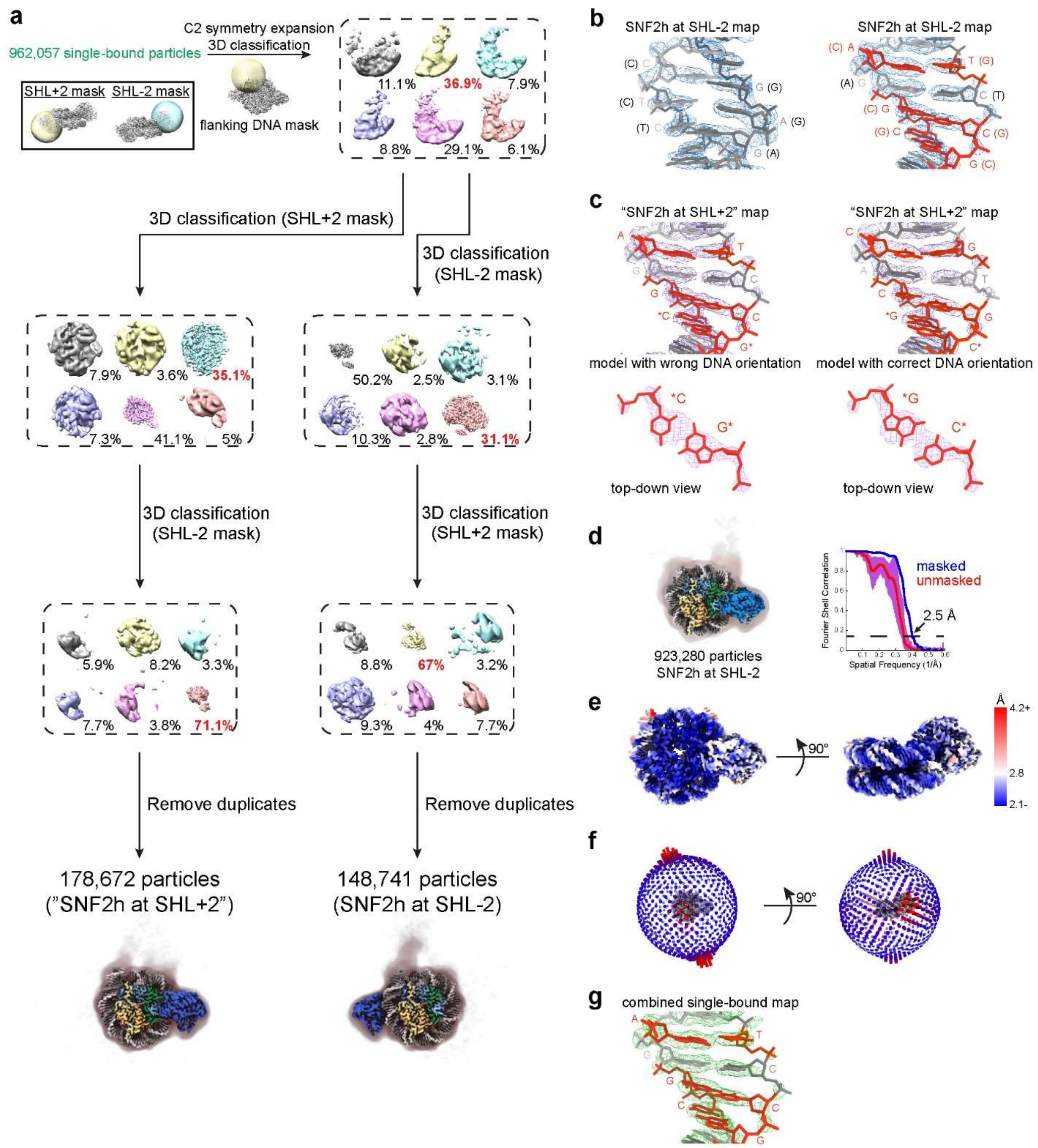

Supplementary Figure 3: Classification and assessment of DNA sequence for single-bound SNF2h-nucleosome complexes.

a) Schematic illustrating processing workflow to identify single-bound SNF2h-nucleosome complexes with SNF2h at either the SHL+2 or SHL-2 position as described in Methods.

b) (left) DNA modeled into region of density in the single-bound SNF2h at SHL-2 nucleosome map corresponding to DNA where purines and pyrimidines remain the same regardless of the orientation of the 601 sequence.

   (right) DNA modeled into region of density in the single-bound SNF2h at SHL-2 nucleosome map corresponding to DNA where certain purines and pyrimidines (in orange) would be flipped depending on the orientation of the 601 sequence. The DNA built with the orientation corresponding to SNF2h at SHL-2 fits the density well.

c) (left) DNA modeled into the same region of density in the single-bound SNF2h at SHL+2 map as in b). The model built with the wrong DNA orientation as in b) does not match the density well. A top view of the CG base pair (marked with asterisks) is shown on the bottom.

   (right) DNA modeled in the opposite orientation into the same region of density in the single-bound SNF2h at SHL+2 map. The model built with DNA in the correct orientation matches the density well, suggesting that SNF2h in this map is at SHL-2 instead.

d) Non-uniform refinement of all 923,280 single-bound particles after duplicate removal resulted in a 2.5 Å global resolution map based on gold-standard FSC determined by cryoSPARC. Directional FSCs determined without masking are in magenta. A Gaussian-filtered map at lower contour is shown as a shadow to indicate the position of flanking DNA, which appears to be conformationally heterogeneous.

e) The single-bound SNF2h-nucleosome map surface colored by local resolution determined by cryoSPARC with FSC cutoff of 0.143.

f) Angular distribution plots for the single-bound SNF2h-nucleosome map.

g) DNA modeled into region of density in the map from a) corresponding to DNA where certain purines and pyrimidines (in orange) would be flipped depending on the orientation of the 601 sequence. The DNA built with the orientation corresponding to SNF2h at SHL-2 fits the density well.

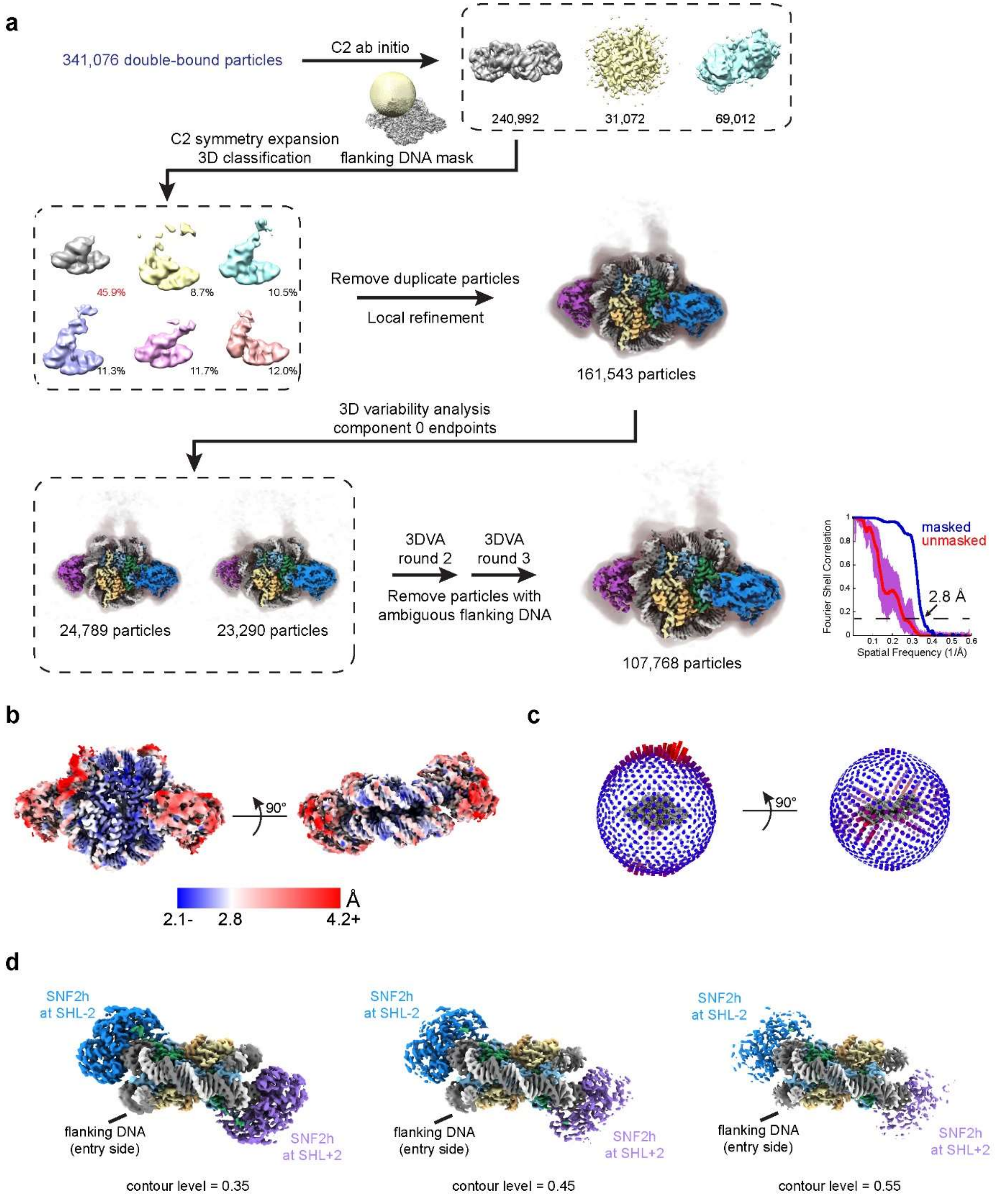

Supplementary Figure 4: Classification for double-bound SNF2h-nucleosome complexes.

a) Schematic illustrating processing workflow to identify the location of flanking DNA for the double-bound SNF2h-nucleosome complex as described in Methods. The gold-standard FSC determined by cryoSPARC is plotted in blue (FSC threshold of 0.143

indicated with dotted line). Directional FSCs determined without masking are plotted in magenta.

b) The double-bound SNF2h-nucleosome map surface colored by local resolution determined by cryoSPARC with FSC cutoff of 0.143.

c) Angular distribution plots for the double-bound SNF2h-nucleosome map.

d) Coulomb potential map of the double-bound SNF2h-nucleosome complex shown at different contour levels. As the contour level increases, the density for SNF2h at the SHL+2 position appears weaker than the density for SNF2h at the SHL-2 position.

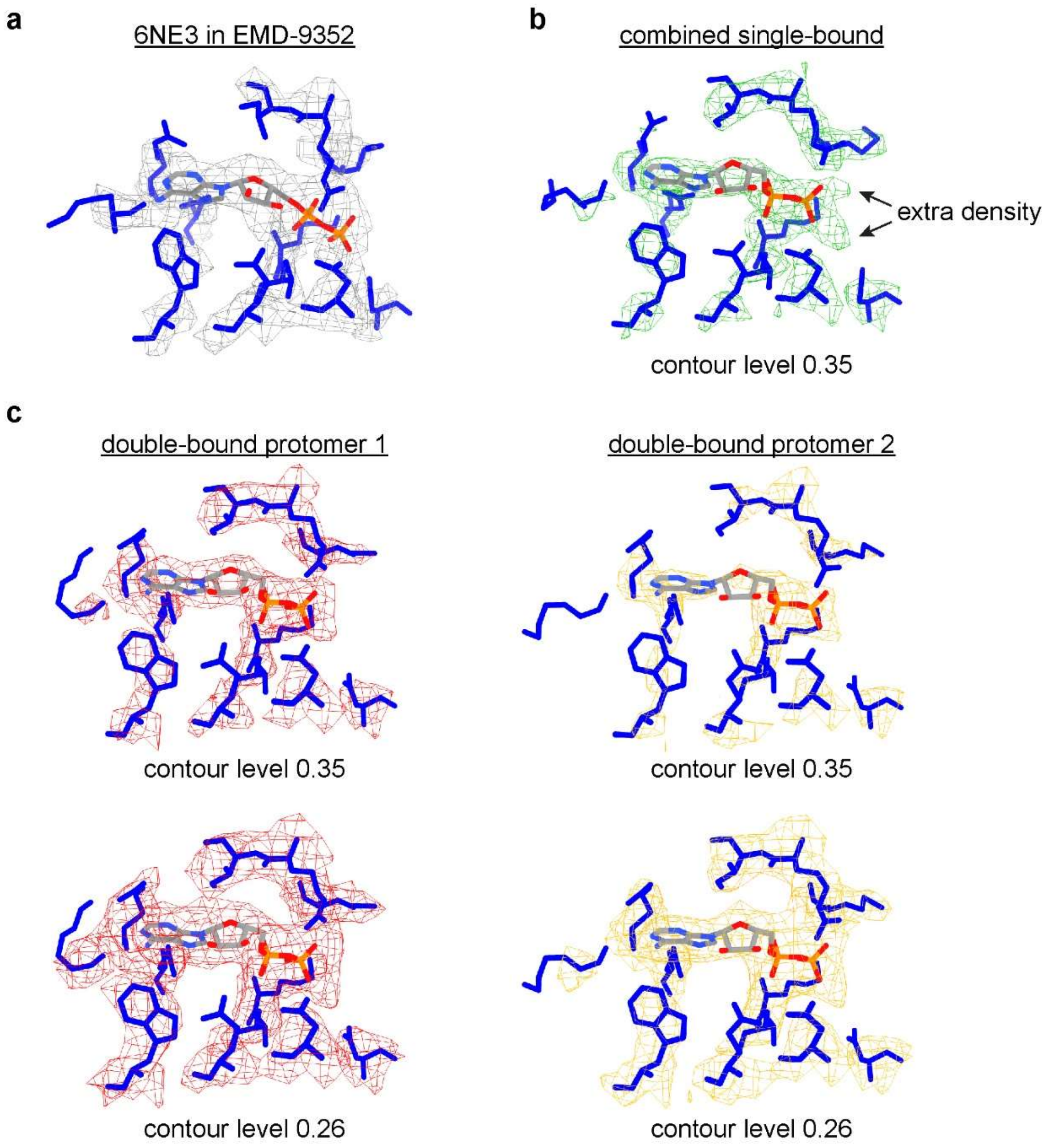

Supplementary Figure 5: Densities for nucleotide in single- and double-bound SNF2h-nucleosome maps.

a) Clear density for only ADP was observed in the previously determined SNF2h-nucleosome structure at 3.4 Å resolution (PDB 6NE3 [https://doi.org/10.2210/pdb6NE3/pdb] in EMD-9352 [https://www.ebi.ac.uk/emdb/EMD-9352]).

b) Extra density corresponding to Mg^{2+} and BeF_x ions are clearly visible in the combined single-bound SNF2h at SHL-2 map.

c) Clear density for only ADP is observed in the SNF2h protomers in the double-bound SNF2h-nucleosome map (density shown at two different contour levels).

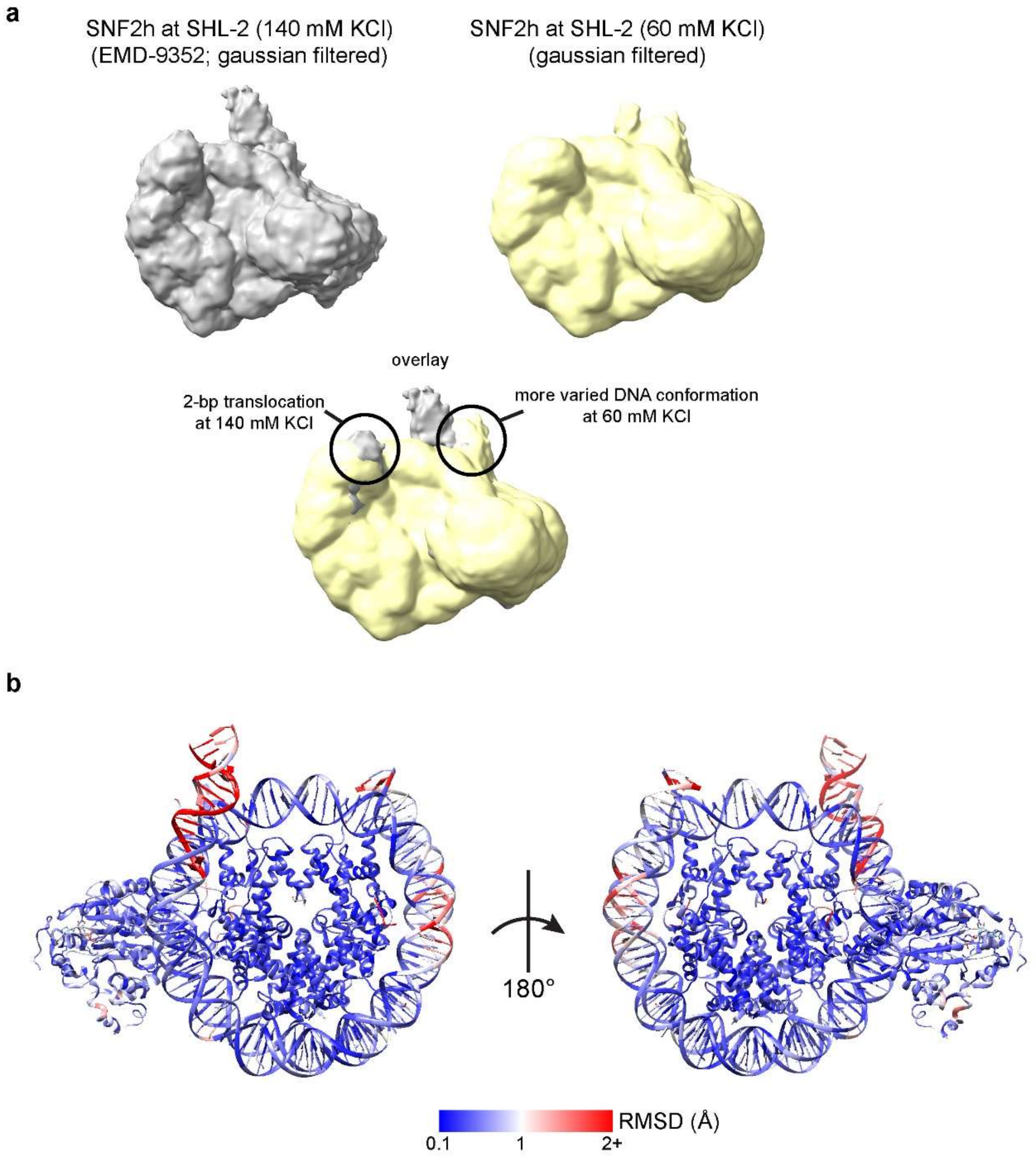

Supplementary Figure 6: Comparison of single-bound SNF2h-nucleosome maps and models.

a) Gaussian filtered maps of the previously determined SNF2h-nucleosome structure at 140 mM KCl (top left; EMD-9352) and of the current SNF2h-nucleosome structure at 60 mM KCl (top right). An overlay of the two maps (bottom) is consistent with a 2-bp translocation observed for the structure at 140 mM KCl but not for the structure at 60 mM KCl. The flanking DNA also displays more conformational variability at 60 mM KCl.

b) Per-residue root-mean-square deviation (RMSD) between the single- and double-bound SNF2h-nucleosome models determined in this study. Most residues within the histone

octamer display RMSD differences of less than 1 Å. Most variation is observed with the flanking DNA and DNA at the SHL+2 position, which is altered due to the binding of the second SNF2h protomer in the double-bound complex.

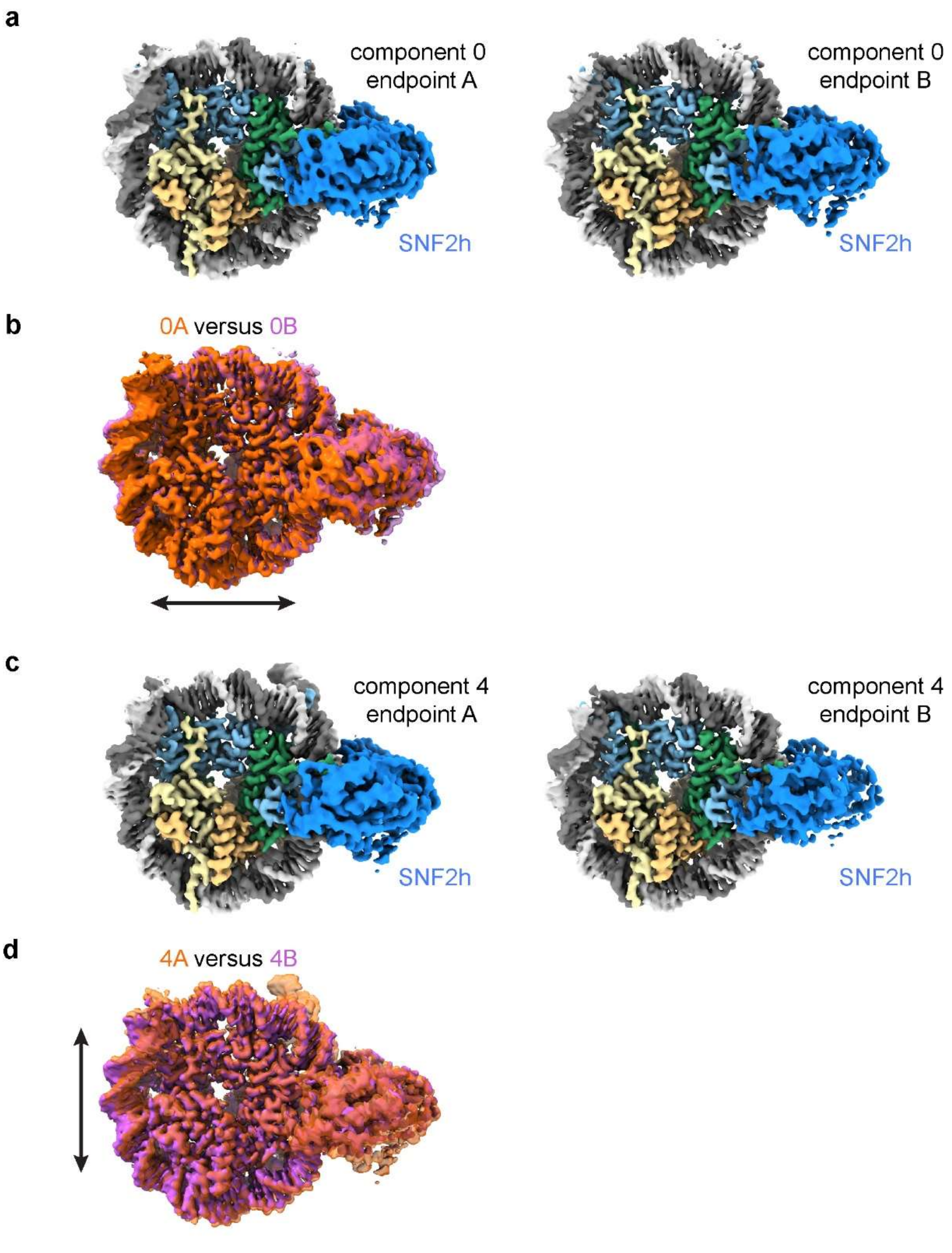

Supplementary Figure 7: Nucleosome expansion and compression.

a) Coulomb potential maps from refinements of subsets of particles corresponding to endpoints of principal component 0 from 3D variability analysis of the single-bound SNF2h-nucleosome particles.

b) Overlay of the maps from a) showing the dilation of structure 0B versus structure 0A in the x direction.
c) Coulomb potential maps from refinements of subsets of particles corresponding to endpoints of principal component 4 from 3D variability analysis of the single-bound SNF2h-nucleosome particles.
d) Overlay of the maps from c) showing the dilation of structure 4A versus structure 4B in the y direction.

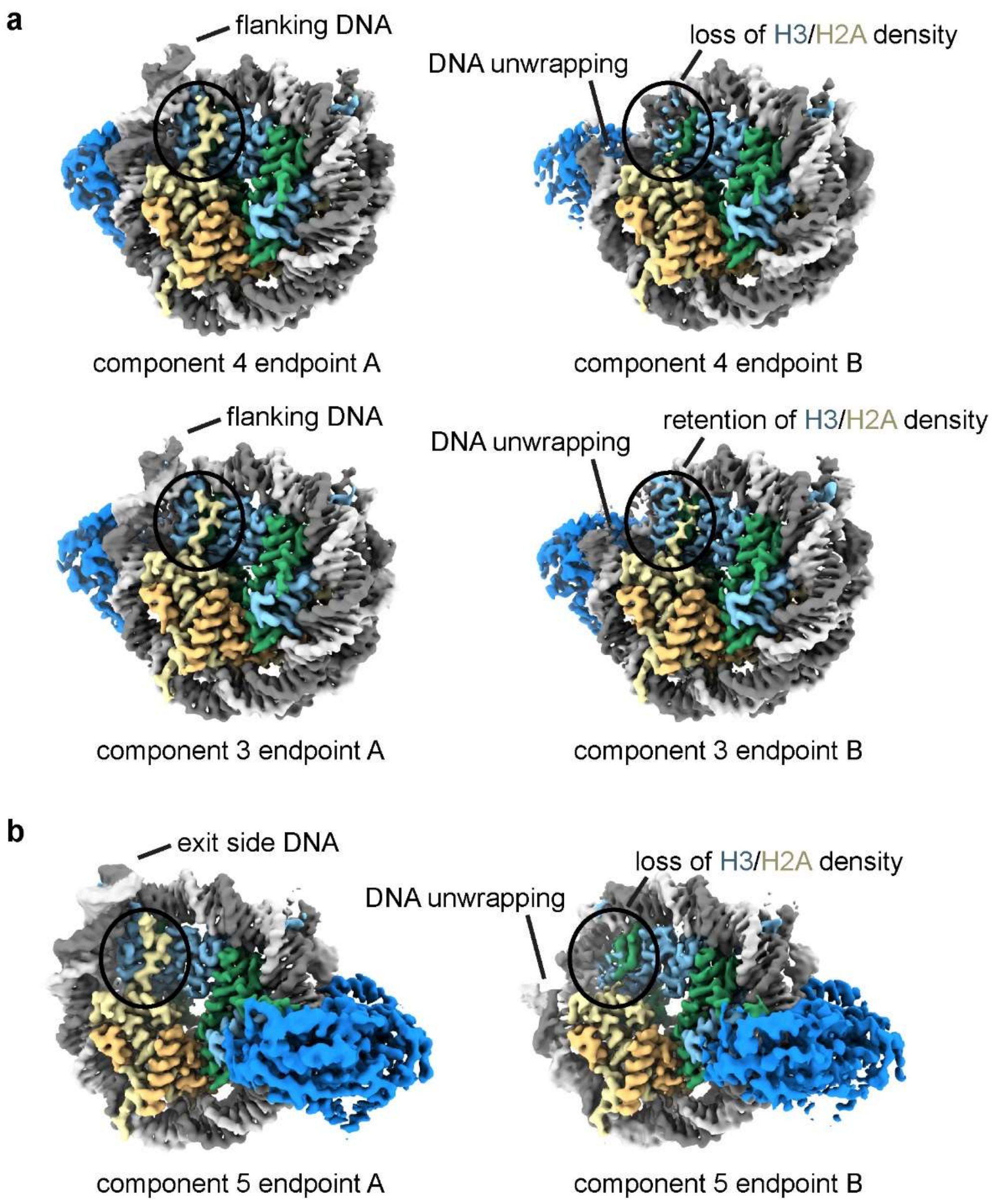

Supplementary Figure 8: DNA unwrapping can occur with both entry and exit side DNA.

a) Coulomb potential maps from refinements of subsets of particles corresponding to endpoints of principal components 3 and 4 from 3D variability analysis of the single-bound SNF2h-nucleosome particles. For these two components, the entry side DNA unwraps, leading to loss of H3 and H2A densities for component 3 but not for component 4.

b) Coulomb potential maps from refinements of subsets of particles corresponding to endpoints of principal component 5 from 3D variability analysis of the single-bound

SNF2h-nucleosome particles. In this component, the exit side DNA unwraps, leading to loss of adjacent H3 and H2A densities.

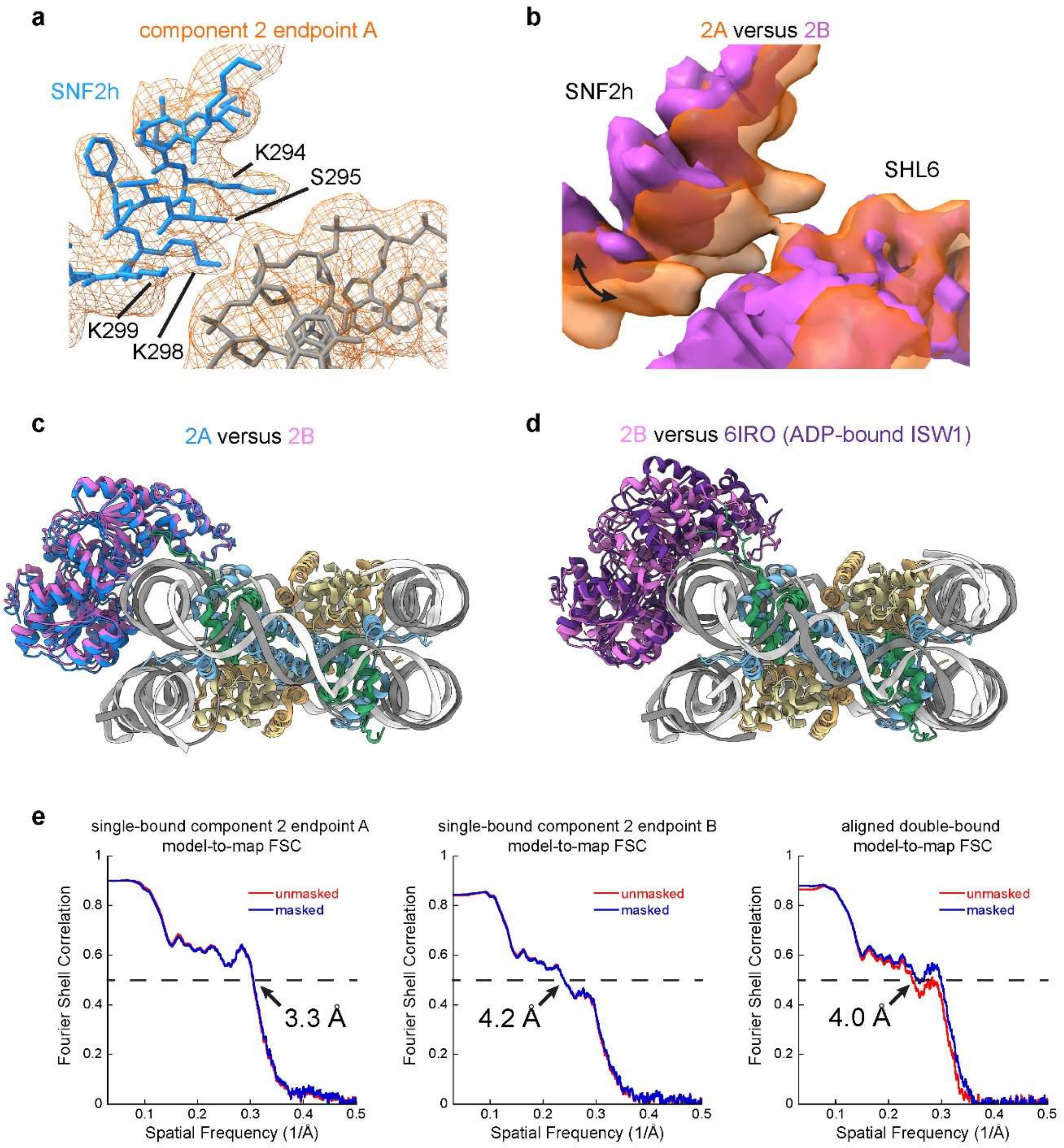

Supplementary Figure 9: SNF2h rocking to and from SHL6.

a) Interaction interface between SNF2h and nucleosomal DNA at SHL6 observed in endpoint structure 2A from 3DVA of the single-bound SNF2h-nucleosome particles. Clear contacts between DNA and residues K294, S295, K298, and K299 of SNF2h are observed.
b) Overlay of coulomb potential maps for endpoint 2A and 2B illustrating movement of SNF2h to and from SHL6.

c) Overlay of the models for single-bound SNF2h-nucleosome complex endpoint structures 2A and 2B. The entire SNF2h ATPase domain rocks upwards in structure 2B (pink) versus structure 2A (blue).

d) Overlay of the models for single-bound SNF2h-nucleosome complex endpoint structure 2B and the previously determined ADP-bound ISW1-nucleosome structure (PDB 6IRO). While the bottom ATPase lobe (lobe 1) appears similarly positioned and dissociated from SHL6, the top ATPase lobe (lobe 2) dramatically shifts upwards in the ADP-bound structure.

e) Model-to-map FSCs for models built in this study.

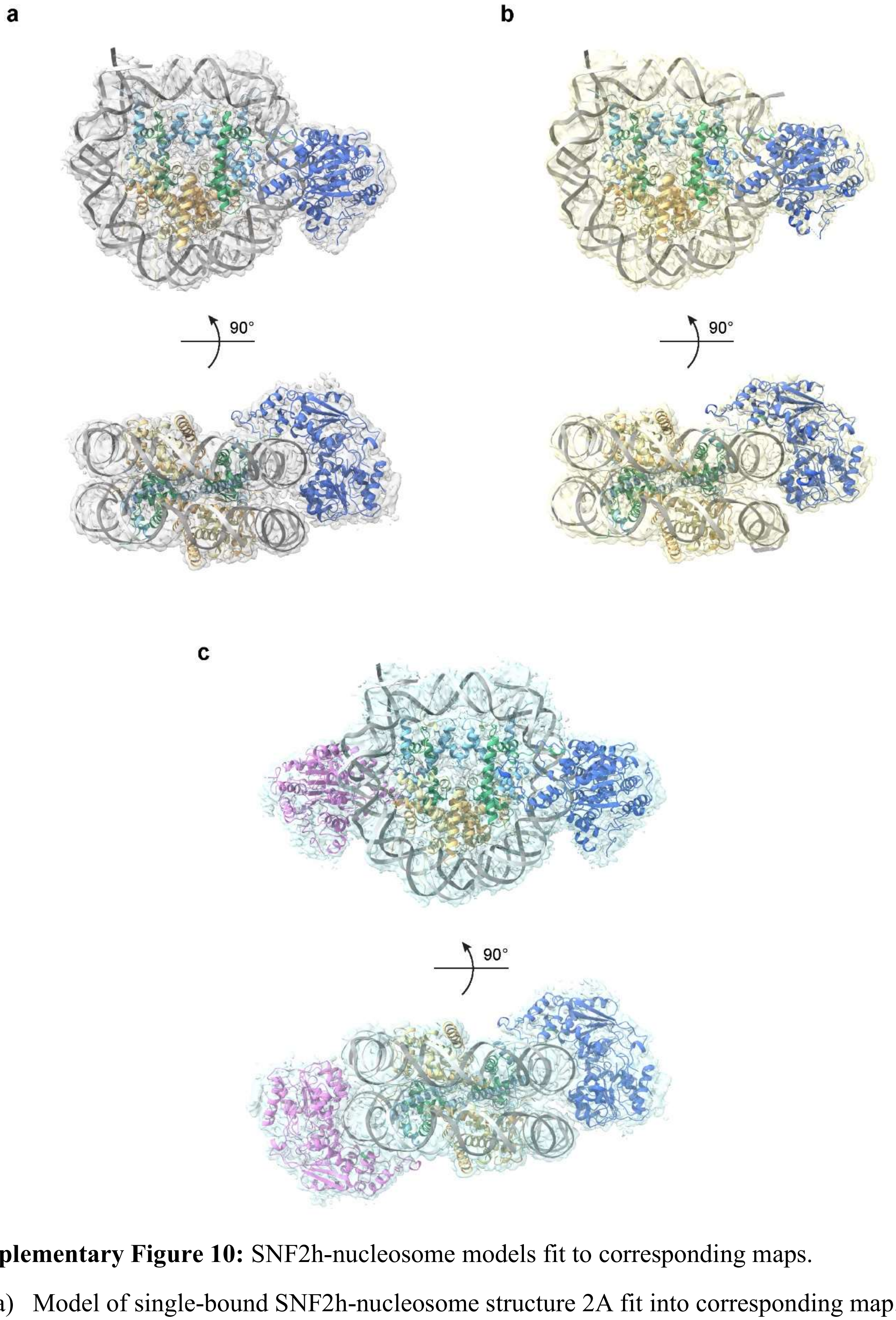

Supplementary Figure 10: SNF2h-nucleosome models fit to corresponding maps.

a) Model of single-bound SNF2h-nucleosome structure 2A fit into corresponding map.

b) Model of single-bound SNF2h-nucleosome structure 2B fit into corresponding map.

c) Model of double-bound SNF2h-nucleosome consensus structure fit into corresponding map.

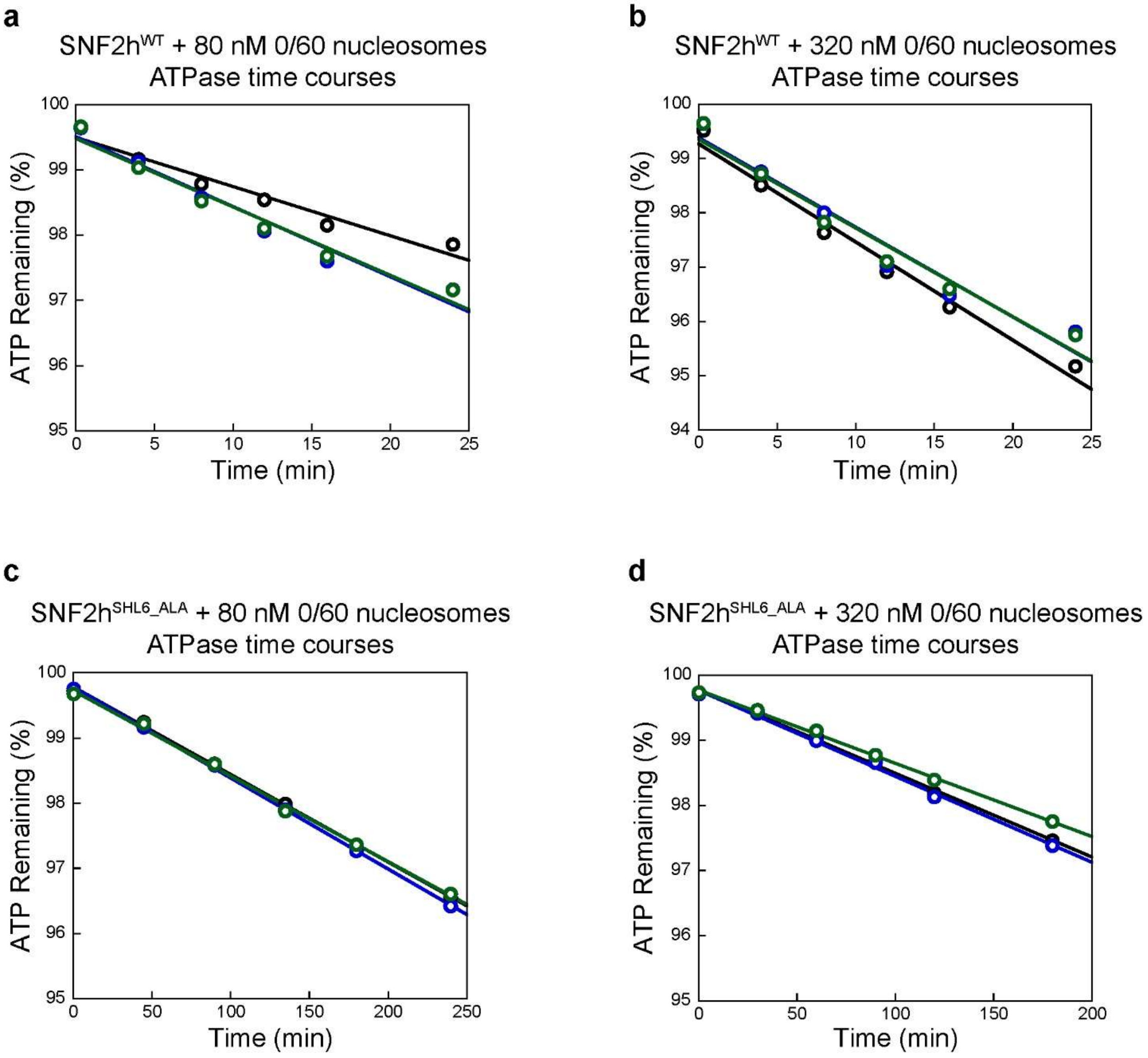

Supplementary Figure 11: ATPase time courses.

Quantified time courses for ATPase assays with (a) 15 nM SNF2h^{WT} and 80 nM nucleosomes, (b) 15 nM SNF2h^{WT} and 320 nM nucleosomes, (c) 15 nM SNF2h^{SHL6_ALA} and 80 nM nucleosomes, and (d) 15 nM SNF2h^{SHL6_ALA} and 320 nM nucleosomes. Each color represents one experimental replicate. Data were fit to a line to determine observed rate constants.

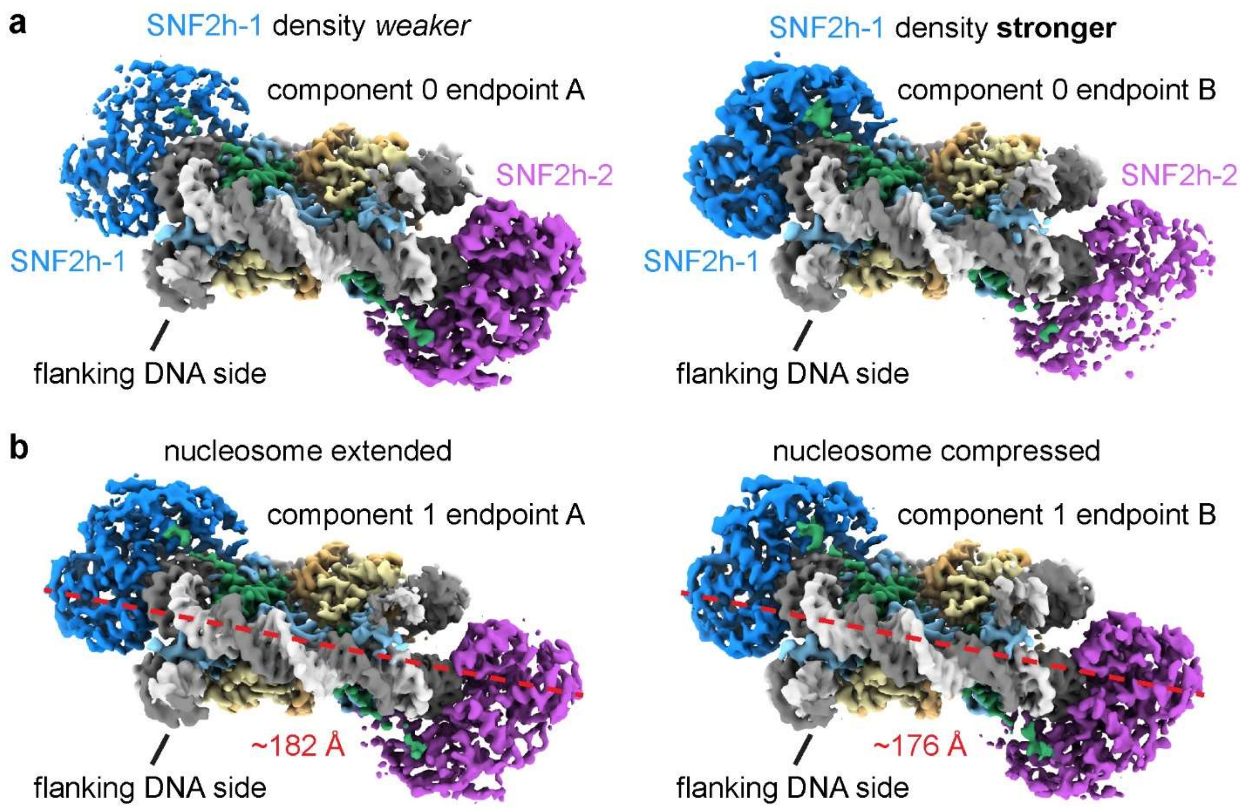

Supplementary Figure 12: Asymmetric and symmetric motions with double-bound SNF2h. Coulomb potential maps from refinements of subsets of particles corresponding to endpoints of (a) principal component 0 and (b) principal component 1 from 3D variability analysis of the double-bound SNF2h-nucleosome particles. In (a), the density for the two SNF2h protomers alternate in strength between the two endpoints. In (b), the nucleosome extends and compresses between the two endpoints.

Supplementary Table 1: Cryo-EM data collection, model refinement and validation statistics

**Data collection and processing**		
Sample	SNF2h-nucleosome (ADP-BeFx)	
Grid type	ssDNA-GO	TAASTY-GO
Microscope	Titan Krios	Titan Krios
Voltage	300 kV	300 kV
Camera	K3	K3
Energy filter	yes	yes
Filter slit width (eV)	20	20
Magnification	105,000	105,000
Pixel size	0.847	0.847
Total electron exposure ($e^{-}/Å^{2}$)	66	66
Defocus range (μm)	(-0.8) - (-2.0)	(-0.8) - (-2.0)
Automation software	SerialEM	SerialEM
Micrographs	7,423	2,020
Particle picker	cryoSPARC template picker	
Total particles extracted	8,509,125	
Particles in initial consensus refinement	1,983,352	

**Reconstruction**	**EMDB 43000**	**43001**	**43002**	**42977**	**43003**	**43004**	**43005**
Subset	consensus	single-bound	double-bound	sb-2A	sb-2B	db-2A	db-2B
Software cryoSPARC	Non-uniform refinement		Local refinement	Non-uniform refinement		Local refinement	
Final particles (dupl. removed)	1,873,513	923,280	107,768	146,702	153,143	16,330	14,666
Symmetry	C1 for all						
Resolution, global (Å)							
FSC 0.5 (unmasked / masked)	3.0 / 2.6	3.2 / 2.8	7.1 / 3.1	6.3 / 3.1	6.8 / 3.2	9.9 / 3.6	10.2 / 3.9
FSC 0.143 (unmasked / masked)	2.7 / 2.3	2.9 / 2.5	4.0 / 2.8	3.2 / 2.8	3.4 / 2.9	7.1 / 3.1	7.3 / 3.2
Local resolution range (Å)	1.87 - 3.64	1.62 - 6.49	1.83 - 5.16	1.83 - 5.87	1.83 - 6.73	1.9 - 8.4	1.95 - 8.1
3DFSC Sphericity	0.968	0.965	0.842	0.651	0.655	0.699	0.634
Sharpening B-factor ($Å^{2}$)	-72.7	-77.5	-56.3	-68.0	-77.5	-27.5	-26.6
**Model Composition**			**PDB 8V6V**	**8V4Y**	**8V7L**		
Protein residues			1673	1219	1203		
Ligands			4	3	2		
DNA			294	294	280		
**Model Refinement**							
Refinement package			Phenix	Phenix	Phenix		
Model-to-map CC			0.68	0.72	0.63		
R.m.s. deviations							
Bond lengths (Å)			0.010	0.010	0.010		
Bond angles (°)			1.505	1.402	1.436		
**Validation**							
Map-to-model FSC 0.5			3.96	3.27	4.17		
Ramachandran (%)							
Outliers			0.18	0.17	0.25		
Allowed			4.24	3.67	3.55		
Favored			95.57	96.16	96.20		
MolProbity score			1.41	1.21	1.49		
Poor rotamers (%)			0.68	0.67	2.22		
Clashscore (all atoms)			3.14	1.88	2.02		
C-beta deviations			0.00	0.00	0.00		
CaBLAM outliers (%)			1.97	1.36	1.81		
EMRinger score			2.91	4.37	3.24		

Supplementary Table 1. Cryo-EM data collection, model refinement, and validation statistics.
