# Supplementary material for: Functionalized graphene-oxide grids enable high-resolution cryo-EM structures of the SNF2h-nucleosome complex without crosslinking
Source: Nat Commun. 2024 Mar 12;15:2225. doi: 10.1038/s41467-024-46178-y (PMC10933330; doi:10.1038/s41467-024-46178-y)
Supplement: Supplementary file 3 — Description of Additional Supplementary Files [file 41467_2024_46178_MOESM3_ESM.pdf]

Description of Additional Supplementary Files

File Name: Supplementary Movie 1
Description: Representative tomogram of SNF2h-nucleosome on ssDNA-GO grid. Movie stepping through slice views at different Z of a representative tomogram of the SNF2h-nucleosome complex on an ssDNA-GO grid. The sample is at a tilt and not perfectly flat. The movie starts from the bottom of the tomogram and then steps to the top. Particles appear along with the appearance of a GO edge or fold closer to the bottom of the tomogram. Crystalline ice contaminants mark the AWI at the top of the tomogram. The movie then boomerangs, stepping through slice views from top to bottom.

File Name: Supplementary Movie 2
Description: Representative tomogram of SNF2h-nucleosome on TAASTY-GO grid. Movie stepping through slice views at different Z of a representative tomogram of the SNF2h-nucleosome complex on a TAASTY-GO grid. The movie starts from the top of the tomogram, where crystalline ice contaminants mark the AWI, and then steps to the bottom. Particles appear along with the appearance of a GO edge or fold closer to the bottom of the tomogram. The movie then boomerangs, stepping through slice views from bottom to top.
