# Supplementary material for: Functionalized graphene-oxide grids enable high-resolution cryo-EM structures of the SNF2h-nucleosome complex without crosslinking
Source: Nat Commun. 2024 Mar 12;15:2225. doi: 10.1038/s41467-024-46178-y (PMC10933330; doi:10.1038/s41467-024-46178-y)
Supplement: Supplementary file 6 — Reporting Summary [file 41467_2024_46178_MOESM6_ESM.pdf]

nature portfolio

Corresponding author(s): Yifan Cheng, Geeta Narlikar, Jean-Paul Armache

Last updated by author(s): Mar 5, 2024



# Reporting Summary

Nature Portfolio wishes to improve the reproducibility of the work that we publish. This form provides structure for consistency and transparency in reporting. For further information on Nature Portfolio policies, see our Editorial Policies and the Editorial Policy Checklist.

## Statistics

For all statistical analyses, confirm that the following items are present in the figure legend, table legend, main text, or Methods section.

| n/a | Confirmed | |
|---|---|---|
| ☐ | ☒ | The exact sample size (*n*) for each experimental group/condition, given as a discrete number and unit of measurement |
| ☐ | ☒ | A statement on whether measurements were taken from distinct samples or whether the same sample was measured repeatedly |
| ☒ | ☐ | The statistical test(s) used AND whether they are one- or two-sided<br>*Only common tests should be described solely by name; describe more complex techniques in the Methods section.* |
| ☒ | ☐ | A description of all covariates tested |
| ☒ | ☐ | A description of any assumptions or corrections, such as tests of normality and adjustment for multiple comparisons |
| ☐ | ☒ | A full description of the statistical parameters including central tendency (e.g. means) or other basic estimates (e.g. regression coefficient) AND variation (e.g. standard deviation) or associated estimates of uncertainty (e.g. confidence intervals) |
| ☒ | ☐ | For null hypothesis testing, the test statistic (e.g. *F*, *t*, *r*) with confidence intervals, effect sizes, degrees of freedom and *P* value noted<br>*Give P values as exact values whenever suitable.* |
| ☒ | ☐ | For Bayesian analysis, information on the choice of priors and Markov chain Monte Carlo settings |
| ☒ | ☐ | For hierarchical and complex designs, identification of the appropriate level for tests and full reporting of outcomes |
| ☒ | ☐ | Estimates of effect sizes (e.g. Cohen's *d*, Pearson's *r*), indicating how they were calculated |

*Our web collection on statistics for biologists contains articles on many of the points above.*

## Software and code

Policy information about availability of computer code

| | |
|---|---|
| Data collection | Serial EM v3.7 or newer was used for cryo-EM data collection. |
| Data analysis | MotionCor2 v1.4.1, cryoSPARC v3.3.2, RELION v3.1, UCSF pyem v0.5, Phenix v1.18.2, COOT v0.9.6, Chimera v1.16.0, ChimeraX v1.4, AreTomo v1.4.2, IsoNet v0.2.1, KaleidaGraph v4.0, ImageQuant TL v8.2 |

For manuscripts utilizing custom algorithms or software that are central to the research but not yet described in published literature, software must be made available to editors and reviewers. We strongly encourage code deposition in a community repository (e.g. GitHub). See the Nature Portfolio guidelines for submitting code & software for further information.

## Data

Policy information about availability of data

All manuscripts must include a data availability statement. This statement should provide the following information, where applicable:

- Accession codes, unique identifiers, or web links for publicly available datasets
- A description of any restrictions on data availability
- For clinical datasets or third party data, please ensure that the statement adheres to our policy

The atomic coordinates generated in this study have been deposited to the RCSB Protein Data Bank under accession codes 8V4Y [https://doi.org/10.2210/pdb8V4Y/pdb] (SNF2h-nucleosome single-bound structure 1), 8V7L [https://doi.org/10.2210/pdb8V7L/pdb] (SNF2h-nucleosome single-bound structure 2), and 8V6V [https://doi.org/10.2210/pdb8V6V/pdb] (SNF2h-nucleosome double-bound structure). The cryo-EM Coulomb potential maps generated in this study have been deposited in the Electron Microscopy Data Bank under accession codes EMD-43000 [https://www.ebi.ac.uk/emdb/EMD-43000] (SNF2h-nucleosome highest resolution consensus map), EMD-43001 [https://www.ebi.ac.uk/emdb/EMD-43001] (SNF2h-nucleosome consensus single-bound map), EMD-42977 [https://www.ebi.ac.uk/emdb/EMD-42977] (SNF2h-nucleosome single-bound map 2A), EMD-43003 [https://www.ebi.ac.uk/emdb/EMD-43003] (SNF2h-nucleosome single-bound map 2B),

EMD-43002 [https://www.ebi.ac.uk/emdb/EMD-43002] (SNF2h-nucleosome consensus double-bound map), EMD-43004 [https://www.ebi.ac.uk/emdb/EMD-43004] (SNF2h-nucleosome double-bound map 2A), and EMD-43005 [https://www.ebi.ac.uk/emdb/EMD-43005] (SNF2h-nucleosome double-bound map 2B). The raw EM movies generated for the SNF2h-nucleosome complex in this study are deposited in EMPIAR under accession code EMPIAR-11909 [https://www.ebi.ac.uk/empiar/EMPIAR-11909]. The ATPase assay and nucleosome remodeling assay data generated in this study are provided in the Source Data file. Source data are provided with this paper.

# Field-specific reporting

Please select the one below that is the best fit for your research. If you are not sure, read the appropriate sections before making your selection.

☒ Life sciences ☐ Behavioural & social sciences ☐ Ecological, evolutionary & environmental sciences

For a reference copy of the document with all sections, see nature.com/documents/nr-reporting-summary-flat.pdf

# Life sciences study design

All studies must disclose on these points even when the disclosure is negative.

| | |
|---|---|
| Sample size | For cryo-EM studies, the data size was limited by available instrument time and relative particle density on cryo-EM grids as described in the EM data processing workflow shown in Supplementary Figure 2. For ATPase assays and nucleosome remodeling assays, experiments were repeated three times for each condition as is standard practice. |
| Data exclusions | Cryo-EM data processing follows standard procedures established for the field and no data was excluded from analysis. No data was excluded for biochemical experiments performed under conditions described in the study. |
| Replication | For ATPase assays and nucleosome remodeling assays, experiments were repeated three times for each condition as is standard practice. Sample preparation for cryo-EM was replicated multiple times (n>3). A large scale cryo-EM data collection was only performed once due to limitations in available instrument time. Full cryo-EM data processing from raw data to final maps was only performed once as is standard practice in cryo-EM structural biology, where the goal is mainly to determine possibly novel structures and replication is typically not done due to limitations in resources and time. |
| Randomization | Resolution is estimated by gold standard Fourier Shell Correlation calculation procedure, where particles are randomly divided by the software (e.g. cryoSPARC v3.3.2) into two subsets. |
| Blinding | No blinding was performed in this study. Blinding is not standard or required in structural biology since there is no bias that can influence structural determination from the raw data. Blinding is also not standard for ATPase and nucleosome remodeling assays since there is no subjectivity in assessment that can lead to biased results. |

# Reporting for specific materials, systems and methods

We require information from authors about some types of materials, experimental systems and methods used in many studies. Here, indicate whether each material, system or method listed is relevant to your study. If you are not sure if a list item applies to your research, read the appropriate section before selecting a response.

## Materials & experimental systems

| n/a | Involved in the study |
|---|---|
| ☒ | ☐ Antibodies |
| ☒ | ☐ Eukaryotic cell lines |
| ☒ | ☐ Palaeontology and archaeology |
| ☒ | ☐ Animals and other organisms |
| ☒ | ☐ Human research participants |
| ☒ | ☐ Clinical data |
| ☒ | ☐ Dual use research of concern |

## Methods

| n/a | Involved in the study |
|---|---|
| ☒ | ☐ ChIP-seq |
| ☒ | ☐ Flow cytometry |
| ☒ | ☐ MRI-based neuroimaging |
